# 

**Published:** 1924-07

**Authors:** 


New Series. Vol. III. No. 34 (Vol. LXXIII, No. 1876). July, 1924. Monthly, Price 6d. (POST FREE 7½d.)

